# In vitro study of the effect of quinoa and quinoa polysaccharides on human gut microbiota

**DOI:** 10.1002/fsn3.2540

**Published:** 2021-08-21

**Authors:** Hitache Zeyneb, Hairun Pei, Xueli Cao, Yuxin Wang, Yumon Win, Lingxiao Gong

**Affiliations:** ^1^ Beijing Advanced Innovation Center for Food Nutrition and Human Health Beijing Technology & Business University Beijing China

**Keywords:** gut microbiota, polysaccharides, Quinoa (*Chenopodium quinoa* Willd.), short‐chain fatty acids (SCFAs)

## Abstract

It has been shown that whole grains and dietary fiber are important for their fermentation characteristics in the large intestine, drawing more and more attention to quinoa and quinoa polysaccharides. In this study, we evaluated the prebiotic effect of quinoa seeds and quinoa polysaccharides after human simulated digestion. The modulatory effect of the quinoa and quinoa polysaccharides (QPs) on the gut microbiota was evaluated by the in vitro fermentation using human fecal microbiota. The yield of polysaccharides extraction was 15.45%. The digestibility of the cooked and uncooked quinoa after simulation of human digestion was 69.04% and 64.09%, respectively. The effect on the microbiota composition and their metabolic products was determined by the assessment of pH, short‐chain fatty acids (SCFAs), and changes in the bacterial population. After 24 hr anaerobic incubation, the total SCFAs of cooked, uncooked quinoa, and quinoa polysaccharides were 82.99, 77.11, and 82.73 mM, respectively with a pH decrease. At the phylum, genus, and class level, it has been found that the quinoa substrates enhance the growth of certain beneficial bacteria such as *Prevotella and Bacteroides*. Quinoa polysaccharides can be considered prebiotic due to their ability to increase *Bifidobacterium* and *Collinsella*. Principal component analysis (PCA) showed that there was a distinct modulating effect on the fecal microbiota which represents different distribution. Our research suggests that quinoa and quinoa polysaccharides have a prebiotic potential due to their association with the positive shifts in microbiota composition and short‐chain fatty acids production, which highlights the importance of further studies around this topic.

## Graphical abstract

Quinoa and quinoa polysaccharides can modulate the gut microbiota. Quinoa and quinoa polysaccharides have a prebiotic effects.

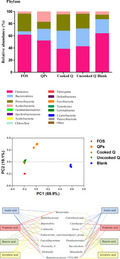

## 1 INTRODUCTION

Quinoa (*Chenopodium quinoa* Willd.) is a native plant in the Andes region of South America, which belongs to the Chenopodiaceae family and a dicotyledonous pseudocereal. More than 6,000 varieties of quinoa are cultivated globally. (Vega‐Galvez et al., 2010). As a traditional cereal, quinoa has been recognized as functional food and nutraceutical due to its important nutritional value and its bioactive components. It has positive effects on human health and is functional to prevent different diseases such as diabetes, cancer, and cardiovascular disease (Hernández‐Ledesma, 2019). Quinoa contains a lot of phytochemicals, including saponins, phytosterols, phytoecdysteroids, phenolic compounds, bioactive peptides, and polysaccharides (Navruz‐Varli & Sanlier, 2016). There is around 10% of dietary fibers in the seeds of quinoa (Lamothe et al., 2015), which is higher than that in rice, corn, and wheat (0.4%, 1.7%, and 2.7% respectively) (Alvarez‐Jubete et al., 2010). In addition, the indigestibility of dietary fibers in the small intestine offers several beneficial effects, such as facilitating the absorption of other nutrients contained in this grain at the level of the large intestine (Ogungbenle, 2003).

Quinoa is a gluten‐free food that can be used to develop new foods for people with celiac disease (Hager et al., 2012). The main carbohydrate component of quinoa is starch, which represents 52%–69% of dry matter (Abugoch James, 2009). Polysaccharides are polymers constituted with the condensation of 10 or more carbohydrate monomers linked together via glycosidic bonds (Chen et al., 2019). Dietary fiber fractions and polysaccharides extracted from quinoa showed a range of biological and functional properties, including anticancer activity, bile acid binding, and radical scavenging activity (Zhu, 2020). In addition, dietary fiber also has beneficial effects on the immune system (Yao et al., 2014). Moreover, the polysaccharides in quinoa seeds demonstrate an anti‐ulcer activity by reducing ethanol‐induced gastric injury and increasing the production of mucus in rats (Cordeiro et al., 2012). Specially, it has been shown that quinoa polysaccharides supplementation may display anti‐hyperlipidemia benefits, which is considered to be a useful active and natural health food for the prevention of hyperlipidemia (Cao et al., 2020).

Human health has always been a topic of concern, particularly with the increase in the incidence rate of metabolic disorders and various diseases. Nowadays, diet seems to be an affordable strategy in the treatment, and prebiotic foods are widely used to prevent and control the regulation of these disorders. Furthermore, both in vivo and in vitro studies have demonstrated that quinoa possesses potential prebiotic effects, with the fact that non‐digestible food ingredient stimulates the activity and/or the growth of one or a limited number of bacteria in the gut, thereby improving host and gastrointestinal health (Gullon et al., 2016). In addition, their consumption inhibited the imbalance of gut microbiota and alleviated the clinical symptoms of colitis induced by dextran sulfate sodium, indicating the potential of quinoa as a dietary method to improve intestinal health (Liu et al., 2018). Herein, this study aimed to determine the regulatory effects of cooked and uncooked quinoa after simulated human digestion and quinoa polysaccharides (QPs) on the gut microbiota. Additionally, we analyzed the metabolites (short‐chain fatty acids (SCFAs)) produced by the human fecal microbial communities during in vitro fermentation. The novel finding of our study may have implications for the design of functional food based on quinoa and quinoa polysaccharides. However, as far as we know, the fermentation of quinoa polysaccharides has not been investigated. The prebiotic properties of this pseudocereal and its polysaccharides need to be further evaluated.

## 2 MATERIALS AND METHODS

### 2.1 Reagents and materials

For digestion of quinoa, different enzymes were used including cellulase (Biotopped, China), α‐amylase (35 units/mg), pancreatin (130 units/g), and bile extract, which were purchased from Shanghai Macklin Biochemical Co. Ltd. Pepsin (3000 units/g) was purchased from Sigma‐Aldrich Shanghai Trading Co. Ltd, China. Diethyl ether, petroleum ether, acetic acid, propionic acid, isobutyric acid, butyric acid, isovaleric acid, valeric acid, and hexanoic acid were purchased from Macklin (AR, Shanghai Macklin Biochemical Co. Ltd, China). Ethanol was bought from National Pharmaceutical (AR, China National Pharmaceutical Group Corporation, China), and distilled water was used. Chromatographic grade methanol used for GC‐MS analysis was purchased from Fisher (HPLC, Fisher scientific, USA). Fructooligosaccharide (FOS) was used as a positive control (Shanghai Macklin Biochemical Co. Ltd, China). Quinoa was purchased from Qinghai Sanjiang fertile ecological agricultural technology Co., Ltd, Haixi prefecture (2018). The quinoa seeds (white quinoa) were ground in an electric mill for 10 s and passed through a sieve to obtain a size between 0.2 and 0.45 mm (80 and 40 mesh). Cooked quinoa was obtained from a standardized home cooking process as follows. 10 g of ground quinoa and distilled water were mixed at a ratio of 1:10 (w/v) in a pot and then heated on a hot plate. During cooking, the temperature of the mixture was kept at 100°C for 20 min with occasional stirring. After cooling, the whole mixture was subjected to a freeze‐drying process.

### 2.2 In vitro digestion of cooked and uncooked quinoa

According to the procedure (Connolly et al., 2010), the simulated digestion was performed with slight modifications. Basically, the cooked and uncooked quinoa were digested in vitro separately, including three different stages: simulated oral, gastric, and small intestine digestion. In the simulated oral digestion, 10 g quinoa was mixed with 50 ml of distilled water and blended for 5 min. α‐amylase was dissolved in the filter‐sterilized aqueous solution of CaCl_2_ (1 mM, pH 7) and then added to the mixture, followed by the incubation at 37°C for 30 min with stirring. In the simulated gastric digestion, the mixture was acidified to pH 2 with HCl (6 M). Pepsin dissolved in HCl (0.1 M) at a ratio of 1:15 (w/v) was added into the mixture and incubated at 37°C for another 2 hr. The pH of the mixture was then adjusted to 6.5 using NaOH (6 M). In the simulated intestinal digestion, 10.4 ml pancreatin (0.33 g) dissolved in NaHCO_3_ (0.5 M, 41.7 ml) and 10.4 ml bile extract (2.08 g) dissolved in NaHCO_3_ (0.5 M, 41.7 ml) were added and the incubation was continued at 37°C for 3 hr with shaking. Finally, the sample solution was transferred into dialysis tubing (Spectra/por 1 kDa MWCO dialysis membrane) and dialyzed overnight with cold sterile NaCl solution (10 mM) at 4°C. After replacement with fresh NaCl solution, the dialysis was continued for another 2 hr. Finally, the dialysate was collected and freeze‐dried.

The digesta yield and digestibility of the samples were calculated by the Equations (1) and (2)

(1) $$]]><InlineMediaObject/><![CDATA[\text{Digesta yield},\%=\frac{W_{1}}{W_{0}}$$

(2) $$]]><InlineMediaObject/><![CDATA[\text{Digesta yield},\%=\frac{W_{0}‐W_{1}}{W_{0}}$$

Where *W_0_* is the initial dry weight of the sample used for enzymatic digestion in vitro and *W_1_* is the dry weight of the residue that could not be digested.

### 2.3 Crude polysaccharides extraction

Quinoa flour was defatted by refluxing with petroleum ether at 60°C for three times to remove pigments and lipids. The ratio of flour to petroleum ether was 1:5. After filtration, the flours were allowed to air‐dry overnight. The extraction of the polysaccharides was carried out by the method (Fan et al., 2019) with a slight modification, where defatted quinoa flour was mixed with distilled water in a ratio of 1:33. The cellulase (enzyme activity 3000 U/g) was added to destroy the cell wall material, and the mixture was heated at 65°C for 1 hr, followed by an ultrasonic extraction at 65°C for 18 min. After extraction, the sample was centrifuged and the supernatant was concentrated to one‐tenth of its volume by vacuum concentration. Anhydrous ethanol was added into the solution at a ratio of 1:3 (v/v) and kept overnight at 4°C to precipitate the polysaccharides. The obtained precipitate was centrifuged and lyophilized for 24 hr to obtain crude polysaccharides.

The deproteinization of quinoa polysaccharides was carried out by the sevage method (Li et al., 2011). The crude polysaccharides were dissolved in distilled water (2 mg/ml), and sevage reagent (dichloromethane: n‐butanol = 4:1) was added at the ratio of 1:2 to remove protein. The solution was shaken vigorously for 20 min and then centrifuged at 3000 *g* for 15 min. The supernatant was collected, and the deproteinization rate (protein absorbance) was determined at 280 nm by spectrophotometer. The deproteinization treatment was repeated until stable absorption was obtained and no white protein layer was observed between the two phases. Then, the sample was dialyzed, concentrated by vacuum concentration, and lyophilized. The polysaccharide extract yield was calculated as follows:

$$\text{Quinoa polysaccharides yield}\;\left(\%\right)=\frac{\text{weight of extracted polysaccharides}}{\text{weight of quinoa}}\times 100$$

### 2.4 In vitro fermentation of different substrates

The fermentation was carried out by the human fecal microbiota. The fecal slurry was prepared as follows: the fecal matter was obtained from a healthy person with a body mass index of 20 kg/m^2^, who declared no previous bowel illnesses. The participant follows a normal diet and has not been treated with antibiotics or probiotics for at least 3 months before fecal sampling. A fresh fecal sample was well mixed with autoclaved phosphate‐buffered saline (PBS, pH 7.0) to extend 10% (w/v) fecal slurry. The mixture was centrifuged at 500 rpm for 5 min, and the supernatant was used for fermentation (Chen et al., 2017).

The following fermentation was conducted in an anaerobic incubator (Thermo SCIENTIFIC), and the anaerobic condition was maintained all the time. The fermentation vessel was filled with a 45 ml sterilized basal growth medium. Each liter of medium contained 2 g Peptone, 2 g Yeast extract, 0.1 g NaCl, 0.04 g K_2_HPO_4_, 0.04 g KH_2_PO_4_, 0.01 g MgSO_4_.7H_2_O, 0.01 g CaCl_2_.6H_2_O, 2 g NaHCO_3_, 2 ml Tween 80, 0.05 g Hemin dissolved in 1 ml of 4 M NaOH, 10 µl vitamin K_1_, 0.5 g L‐Cysteine HCL, and 0.5 g Bile Salts (sodium glycocholate and sodium taurocholate). The medium was adjusted to pH 7.0, and 4 ml of 0.025% (w/v) resazurin solution was added before autoclaving (121°C, 30 min). Once in the fermentation vessels, the sterile medium was sparged with N_2_ (15 ml/min) to maintain anaerobic conditions. To test the substrates, 1 g of digested cooked and uncooked quinoa, quinoa polysaccharides and the positive control fructooligosaccharides, were added to the basal medium respectively, giving a final concentration of 1% (w/v) before inoculating 5 ml of pretreated fecal supernatant with the basal medium sample without substrate as blank. The fermentation was maintained at 37°C for 24 hr.

The change of pH value was measured by a pH meter at different times of 0, 6, 12, and 24 hr. To stop the fermentation, the fermentation products (sampling) were extracted into vials and kept in ice water for 20 min and the final pH value was represented by the mean and standard deviation of three measurements.

### 2.5 Analysis of short‐chain fatty acids

Short‐chain fatty acids (SCFAs) are the main metabolites produced by the fecal microbiota. After 24 hr of fermentation, the sample was centrifuged at 13,000 × *g* for 5 min. 5 ml sample was acidified with 0.3 ml sulfuric acid (7.1 mol/L) in a 10 ml centrifuge tube. The short chains fatty acids were extracted with 3 ml cold diethyl ether with shaking vigorously for 10 min and then centrifuged. The extraction was repeated three times. All the upper phases were combined and filtrated before GC‐MS analysis.

Qualification and quantification of short fatty acids were determined using a Shimadzu (GC‐2010) gas chromatography coupled with a Shimadzu MS detector (MS‐2010). The carrier gas was helium with a flow rate of 3 ml/min. The injection port was used with a split mode at 250°C and the split ratio was 30. The chemical separation was performed on an INNOWAX capillary column (30 m × 0.32 mm ×0.25 μm, Agilent Technology, USA). The oven temperature was set initially at 80°C followed by an increase of 5°C/min to 150°C. Then, it was increased to 230°C at the rate of 25°C/min and maintained for another 2 min. The MS detector was operating in an electron ionization (EI) voltage of 70 eV under a mass scan range of 33–350 m/z. The temperature of the interface and the ion source was set at 230°C. The concentrations of SCFAs were calculated through an external standard curve of a mixture of acetic acid, propionic acid, isobutyric acid, butyric acid, isovaleric acid, valeric acid, and hexanoic acid.

### 2.6 Microbiota analysis by 16S rRNA gene sequencing

The 0 and 24 hr fermentation products were sampled and stored at −80°C for microbiota analysis. Genomic DNA was extracted using a DNeasy PowerSoil Kit (QIAGEN) following the manufacturer's instructions. The metagenomic analysis was carried out in Beijing Laike Biotechnology Company (China). Primers 343F‐5′‐ TACGGRAGGCAGCAG ‐3′ and 798R‐5′‐ AGGGTATCTAATCCT‐3′ were used to construct the 16sRNA V4 library. The rest high‐quality 16sRNA sequence was pre‐clustered into operational taxonomic units (OTUs) with a 97% similarity cutoff by Vsearch (version 2‐4‐2). The RDP classifier was used to annotate the representative sequence found in each OTU and blaste against Silva database Version 132 (Or Greengens).

The taxonomy of each 16S rRNA gene sequence was analyzed by the RDP Classifier algorithm. Alpha diversity analysis evaluates species richness and distribution uniformity in samples by calculating different alpha diversity indices based on the diversity index and using the alpha diversity boxplot analysis (Kruskal–Wallis/Wilcoxon and other algorithms).

### 2.7 Statistical analysis

All values are presented as mean ± standard deviation (*SD*) of triplicate analysis. Statistica software (version 5.5.fr; StatSoft, Inc, Tulsa, USA) was used to analyze the results. One‐way analysis of variance (ANOVA) following the LSD test (least significant difference) was used to determine significant differences (*p* < .05) among the means. Pearson's correlation between gut microbiota and the short‐chain fatty acids produced and the corresponding *p*‐value was calculated.

## 3 RESULTS AND DISCUSSIONS

### 3.1 Cooked and uncooked quinoa digestibility

**FIGURE 1:**
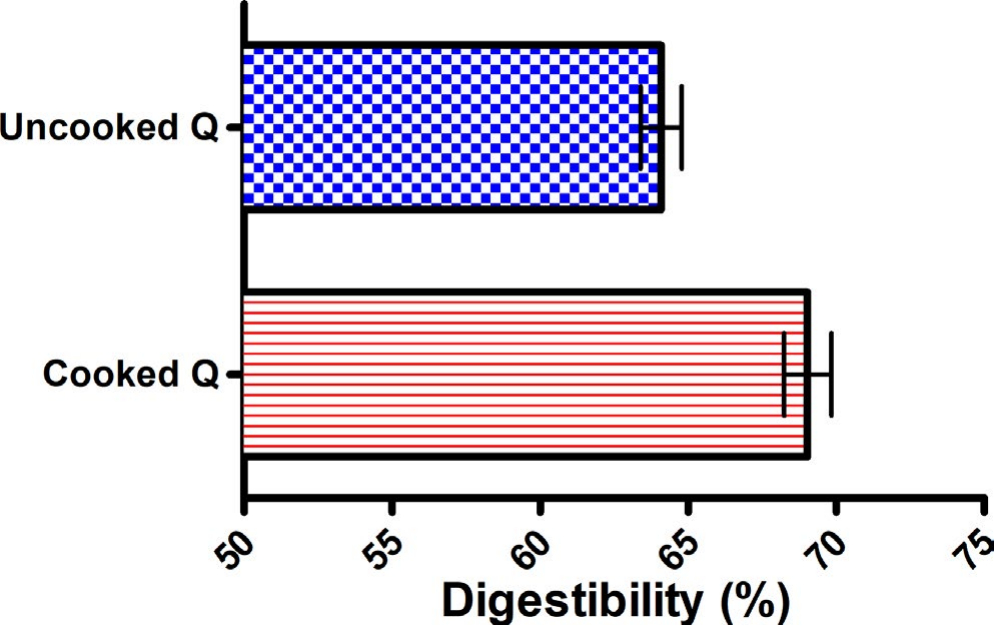
The digestibility of cooked and uncooked quinoa

This section aimed to compare the effects of raw and cooked grains on intestinal health and whether home cooking affected the grains. The digestibility yield of cooked and uncooked quinoa was 69.04% and 64.09%, respectively (Figure 1), and the digestibility of cooked quinoa was higher than that of uncooked quinoa. It is well known that cooking can modify the composition of food in different ways and have a certain effect on the human body and gut microbiota composition (Perez‐Burillo et al., 2018). After digestion, the yield of the undigested matter of the uncooked seeds was higher than that of the cooked seeds, indicating that the uncooked seeds were resistant to enzymatic degradation and provide more substrates to be fermented by the gut microbiota. Cooking serves to facilitate the digestion of seeds and make them easier to be digested. Cooking can change the physicochemical characteristics of cereals due to the combination of humidity and high temperature, which can increase the glycemic index of certain cereals as well and modulate certain properties such as the enzymatic inhibitory property and the antioxidant activity (Adedayo et al., 2018). It was found that grains are widely preferred after processing to be incorporated into consumption due to their high bioavailability, which is why quinoa grains have been incorporated into various formulas such as bread, gluten‐free formulas, or bread‐based on rice and potato (Ikram et al., 2021).

### 3.2 Polysaccharides extraction

The yield of polysaccharides extracted by the ultrasonic was 15.45 ± 0.08% of dry weight, with the deproteinization rate of 71.52 ± 0.9%. The deproteinization procedure was the fundamental step in the analysis of natural plant polysaccharides. According to a previous study, the proteins cannot be completely eliminated due to the existence of proteoglycan and glycoproteins and the intensive binding between polysaccharides and proteins (Chen et al., 2012). In addition, the removal of proteins with this method repeatedly led to a loss of the polysaccharides. The final extraction rate of polysaccharides decreased to 7.69 ± 2.10%, which was reduced by 49.74 ± 1.44%. The content of carbohydrates in quinoa polysaccharides was 56.30% before deproteinization and increased to 90.08% after the deproteinization process.

### 3.3 pH changes during fermentation

**FIGURE 2:**
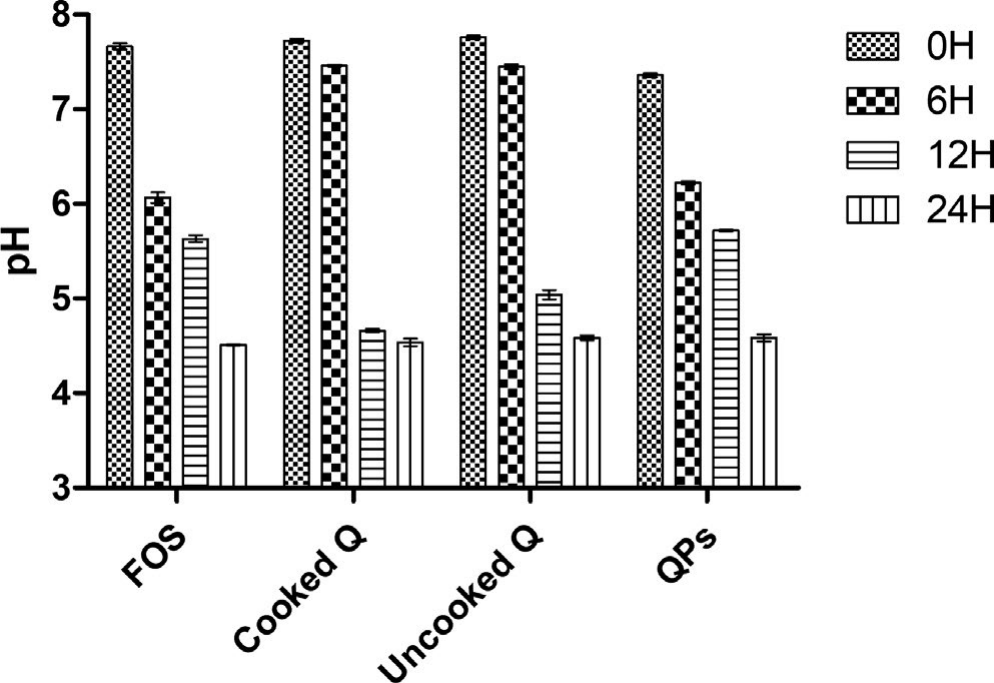
pH changes during the fermentation of the different substrates

The change of pH is the main index of fermentation. As shown in Figure 2, the initial pH of the following fermentation substrates, cooked quinoa, uncooked quinoa, quinoa polysaccharides, and fructooligosaccharides, was 7.72, 7.76, 7.36, and 7.66, respectively. The pH decreased in all fermentation samples over time. The short‐chain fatty acids were produced in the fermentation process, which can reduce the pH, increase the solubility of some minerals, promote passive absorption, and inhibit the growth of certain unsafe bacteria (Peredo‐Lovillo et al., 2020). Among the main determinants of the final acid production after fermentation, butyric acid is in the moderate acidic range (pH 5.5) (Walker et al., 2005), and propionic acid is chosen by enterocytes as a favorable energy source after butyric acid (Bilotta et al., 2021). These acids play an important role in the treatment of inflammatory bowel diseases because of their anti‐inflammatory properties (Tedelind et al., 2007).

Furthermore, it has been revealed that acidic pH improves gut health by developing the gut microenvironment and restraining the pathogenic bacteria growth (Slavin, 2013). There are several substrates suitable for the growth of human intestinal microbiota, including oligosaccharides and dietary fibers. The fermentation of these substrates serves to acidify the intestinal medium by producing fatty acids, which can regulate the cellular process and serve as fuel (Blaut, 2002). This is why the decrease of pH can help us predict the nutritive effect of quinoa on the gut cells, and leads to beneficial effects on human intestinal health.

### 3.4 Short‐chain fatty acids production

Intestinal microorganisms produce essential short‐chain fatty acids for the human body by fermenting food in the colon. Fatty acids provide energy for cells, affect colon metabolism, and control the proliferation and variation of epithelial cells (Rios‐Covian et al., 2016). The short‐chain fatty acid concentration of the different test substrates after 24 hr fermentation was summarized in Table 1. The results showed that there were significant differences (*p* < .05) between the different substrates. Propionic acid and butyric acid were detected as the predominant acids in the fermentation slurries of cooked and uncooked quinoa, while butyric acid and valeric acid were the main acids in quinoa polysaccharides, similar to FOS. Meanwhile, acetic acid was found in all quinoa substrates but not in FOS. The lack of some acids in some media might be due to the rapidity of metabolization of these acids by the gut microbiota and the specificity of the production of acids by the colon bacteria (Yang & Zhao, 2021). The reason remains to be explored further. These results showed that the substrates used were metabolized by several bacteria in the fecal microbiota.

**TABLE 1:** SCFAs content in fermentation production of different substrates (mM)

	Acetic acid	Propionic acid	Isobutyric acid	Butyric acid	Isovaleric acid	Valeric acid	Hexanoic acid	Total acids
Cooked	9.82 ± 0.13^a^	36.03 ± 0.51^a^	ND	22.49 ± 0.73^c^	1.71 ± 0.01^b^	13.92 ± 0.18^c^	ND	82.99 ± 1.82^a^
Uncooked	9.76 ± 0.09^a^	37.08 ± 0.59^a^	ND	18.11 ± 0.59^d^	1.59 ± 0.02^c^	11.45 ± 0.46^d^	ND	77.11 ± 1.87^c^
QPs	9.26 ± 0.72^a^	15.09 ± 0.79^b^	1.77 ± 0.04	37.15 ± 0.41^a^	2.30 ± 0.06^a^	16.29 ± 0.60^b^	ND	82.73 ± 2.55^a^
FOS	ND	5.80 ± 0.06^c^	ND	25.20 ± 0.39^b^	1.59 ± 0.01^c^	23.85 ± 0.50^a^	7.55 ± 0.08	63.98 ± 0.86^d^

Note

Results in each column are statistically different (ANOVA, LSD test, *p* < .05) with a > b > c > d.

Abbreviations: FOS, Fructooligosaccharide; ND, not detected; QPs, quinoa polysaccharides.

Quinoa polysaccharide has shown anti‐hyperlipidemia benefits (Cao et al., 2020). A previous study showed that propionate, butyrate, and acetate produced by the fecal microbiota during anaerobic fermentation were transported to the liver and other tissues for metabolism, which can have a hypolipidemic effect and prevent cardiovascular disease. These acids regulate the expression and the metabolite pathways of nine key genes associated with the biosynthesis of intestinal cholesterol. In addition, it turned out that short‐chain fatty acids have nutritional effects on the intestinal mucosa and colonic epithelial cells (Alvaro et al., 2008). Also, the SCFAs produced during carbohydrates fermentation increased the production of glucagon‐like peptide 1 (GLP‐1) and peptide YY (PYY), which reduces both the concentration of low‐density lipoprotein (LDL) cholesterol in the blood and the accumulation of free fatty acids in the liver, helping to reduce the hepatic steatosis (Surampudi et al., 2016). Quinoa polysaccharides have also been shown to produce butyric acid. This butyrogenic effect is the result of cross interactions between Bifidobacteria, which are anaerobic bacteria belonging to the phylum Actinobacteria, and butyrate‐producing colon bacteria, which produce butyric acid from the metabolite of bifidobacterial strain (such as lactate or acetate) in the human colon (Riviere et al., 2016).

### 3.5 Analysis of microbial communities with different substrates

**FIGURE 3:**
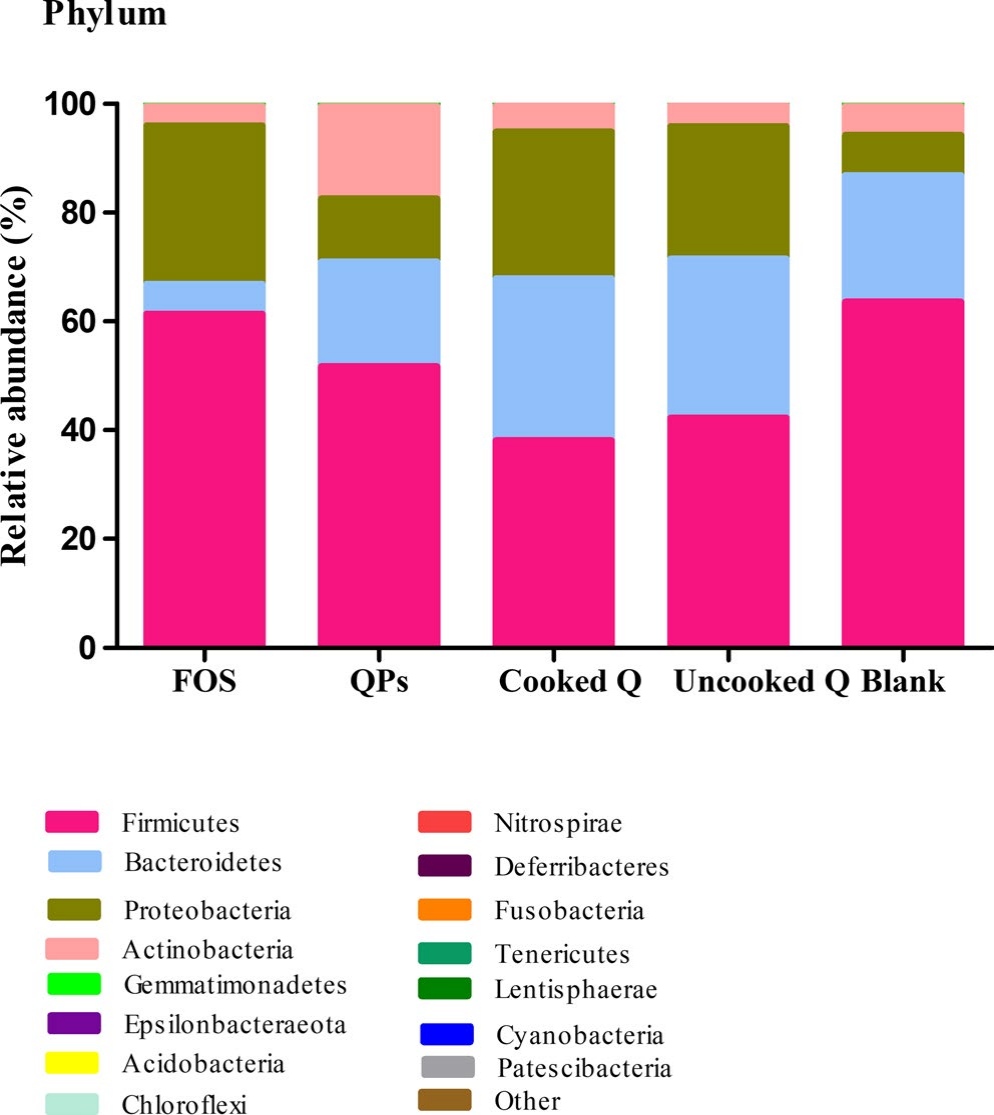
Relative abundances of gut microbial phylum before and after fermentation. QPs, quinoa polysaccharides; FOS, Fructooligosaccharide; Blank, before fermentation. Results are expressed as the average value of triplicates

The changes of microbial communities in fecal samples fermented with different substrates were shown in Figure 3. The microbiota after fermentation with the substrates was different from that of the initial microbiome. Before fermentation, Firmicutes and Bacteroidetes were detected as the dominant phyla in all fermentation samples (63.53% and 23.28%, respectively). After 24 hr of fermentation, the relative abundance of Firmicutes had decreased significantly to 38.07%–61.33%. For Bacteroidetes, the relative abundance of cooked and uncooked quinoa was increased to 29.74% and 29.25%, respectively. Except for quinoa polysaccharides and FOS, their contents decreased to 19.24% and 5.51%, respectively. In the control group, actinobacteria accounted for 5.28%, which increased to 16.98% in the culture of quinoa polysaccharides. The phylum Actinobacteria and Bacteroidetes were considered to be beneficial bacteria, which had an important function in the development of the surface of epithelial cells and the maintenance of homeostasis (including protection and nutrition) (Barczynska et al., 2016). While the relative abundance of Proteobacteria for all the substrates increased from 7.39% to 11.6%–29.08%, it contributes to the homeostasis of anaerobic bacteria in the intestine and the stability of the strict anaerobic microbiota required for appropriate intestinal functioning (Shin et al., 2015), as well as due to the microbial composition of the donor (Perez‐Burillo et al., 2018).

**FIGURE 4:**
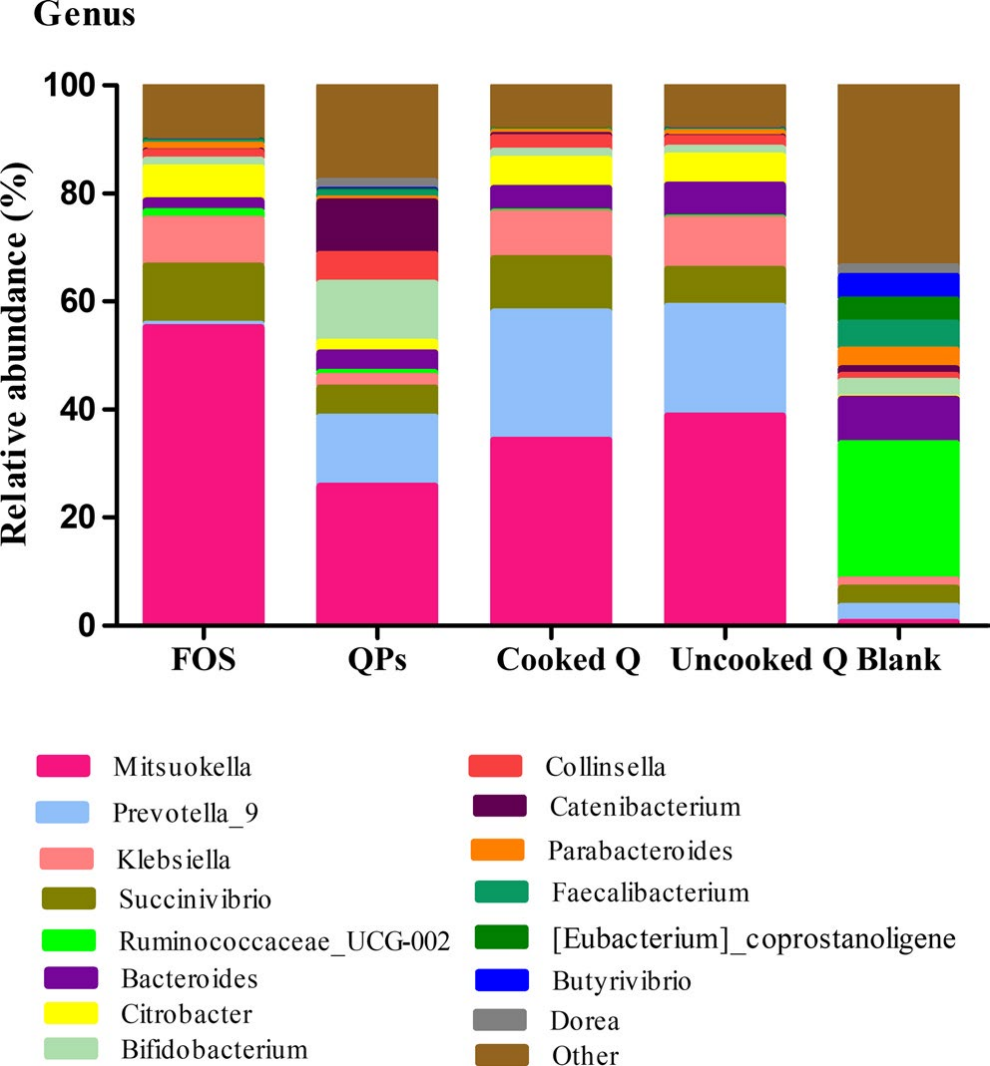
Relative abundances of gut microbial genus before and after fermentation. QPs, quinoa polysaccharides; FOS, fructooligosaccharide; Blank, before fermentation. Results are expressed as the average value of triplicates

**FIGURE 5:**
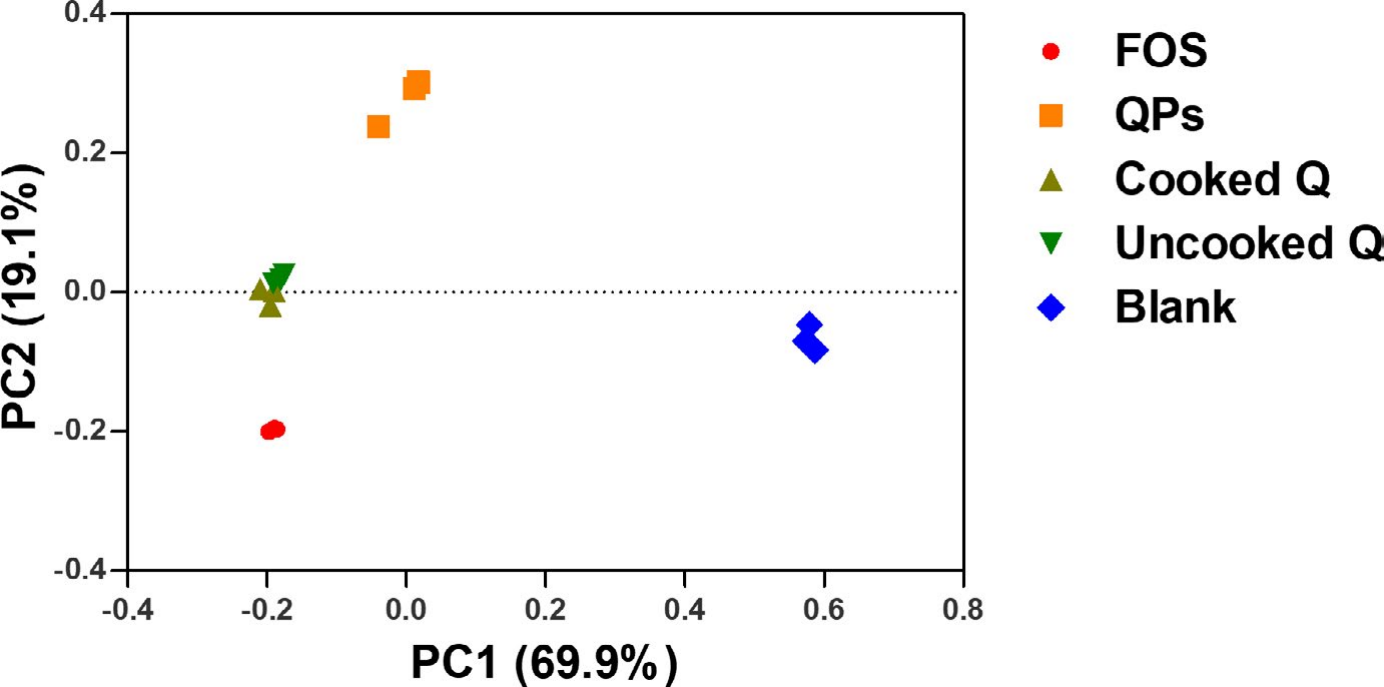
Principal component analysis of microbial 16S rRNA sequences from the V4 region in fermentation slurry with cooked, uncooked quinoa, QPs quinoa polysaccharides, FOS fructooligosaccharide as a positive control for 24 hr and blank before fermentation

The differences between cooked, uncooked quinoa, quinoa polysaccharides, FOS as the positive control and blank were shown in Figures 4 and 5. We found that the microbial composition was diversified at the phylum and genus level. Quinoa substrates (cooked quinoa, uncooked quinoa, and quinoa polysaccharides) promote the growth of some beneficial bacteria after fermentation, such as *Bifidobacterium* and *Collinsella*. The difference in comparison to the FOS may be due to the composition of carbohydrate. The differences of microorganisms between cooked, uncooked quinoa, and quinoa polysaccharides could be explained by the fact that polysaccharides can be metabolized easily by fecal microorganisms and depends on the bioavailability of polysaccharides to colonic microbiota after intake. This means that its chemical composition was carbohydrate complex, while cooked and uncooked quinoa grains contain different components that could interfere with the colonic fermentation process.

In the fecal samples fermented with cooked quinoa and uncooked quinoa, there was no difference in the microbial communities of some phyla, such as Actinobacteria and Bacteroides, whereas there was a significant difference in the microbial communities of other phyla, such as Firmicutes and Proteobacteria. Although the amounts of acids produced were different, the points of difference may be due to cooking. This treatment helped to weaken the structure of seeds. It was previously mentioned that the soluble fraction of quinoa fibers increases during cooking since this treatment increases their solubilization (Zhu, 2020). Furthermore, Perez‐Burillo et al. discovered that the Maillard reaction in some thermal treatment processes provides fiber‐like substrates, such as melanoidins, which could promote the growth of a certain type of bacteria (Perez‐Burillo et al., 2018). In different fermentation substrates, the quinoa polysaccharides offered various microbial structures, while the cooked quinoa provided a high amount of fermentation metabolites. However, not all the substrates showed the same prebiotic effect, which was related to several factors. Several studies have found that dietary fibers which are polysaccharides from various sources affect in different ways the composition of colon bacteria and SCFA production, which improve human gut health and immune function (Yang & Zhao, 2021). Furthermore, dietary fiber reduces transit time and increases satiety and intestinal motility. In addition to the immunomodulatory effects of polysaccharides, specifically arabinoxylan, a previous study showed that they regulate the level of lipopolysaccharides in the blood in the case of high‐fat diets (Li et al., 2019). Moreover, inulin‐type fructan Wich is a prebiotic polysaccharide that increases HDL cholesterol, decreases LDL cholesterol, and lowers blood glucose and triglycerides in diabetics patients (Li et al., 2021).

At the genus level, 15 bacteria genera were detected in the fecal slurries (Figure 4). Compared with the control group, a remarkable increase was observed for the following genus including *Mitsuokella* increased from 0.62% to 25.8%–55.19%, *Succinivibrio* from 3.32% to 5.38%–10.76%, *Klebsiella* from 1.59% to 2.15%–9.2%, *Citrobacter* increased from 0.22% to 2.02%–6.13%, and *Prevotella* from 2.98% to 12.78%–23.68%, except the FOS. The increase of *Klebsiella* in the cooked, uncooked quinoa, and the positive control may be due to the existence of certain fermentable compounds. HPAEC analysis showed that xylobiose, triose, and tetraose were fermentable compounds of *Klebsiella* (Van Laere et al., 2000). As shown in a previous study, a diet rich in fiber was associated with an increase in *Prevotella* abundance, providing an explanation that the increase of the *Prevotella* genus is probably due to the fermentation of fibers (Kovatcheva‐Datchary et al., 2015). This genus, which belongs to the *Prevotellaceae* family, is known for its role in the production of SCFA (Yang & Zhao, 2021). *Collinsella* was also increased after fermentation of all substrates, which was related to the decreased severity or incidence of multiple diseases (such as diabetes, cancer, obesity, inflammatory bowel disease, etc.) (Perez‐Burillo et al., 2018).

In the polysaccharides group, *Bifidobacterium* increased from 3.33% to 10.88%, whereas the abundance of the following genera decreased, such as *Bacteroides* decreased from 8.17% to 1.93%–6.08% in the blank, *Parbacteroides* decreased from 3.50% to 0.60%–1.28%, *Eubacterium* decreased from 4.29% to 0.26%–0.02%, *Faecalibacterium* decreased from 4.95% to 1%–0.26% and *Ruminoccaceae –UCG‐002* decreased from 25.13% to 1.43%–0.23%. Our results suggested that quinoa polysaccharides, cooked quinoa, and uncooked quinoa have a significant difference in the regulation of gut microbiota. It is shown that the polysaccharides could be used as a crude source of carbohydrate for fermentation, indicating that quinoa polysaccharides had an obvious prebiotic promoting effect on the *Bifidobacterium* and other probiotics. Based on the finding of Fukuda et al., (2011), *Bifidobacterium* is an important gut bacterium, which had many important physiological functions availing to human health, such as synthesis of certain vitamins as (vit K, vit B) and enhancing the absorption of minerals like Ca^++^ and Fe^++^. Furthermore, *Bifidobacterium* contributes to the reduction of type I diabetes and colorectal cancer (Perez‐Burillo et al., 2018). It was found that the food intake can affect the abundance of *Bifidobacterium* in the gastrointestinal tract, and age can also affect its abundance. Therefore, there is more *Bifidobacterium* in infants than adults, and its abundance varies from one person to another even if their age is similar (Yang et al., 2021). Bifidobacteria could activate the immune system response by enhancing the production of interleukin 6 (IL‐6) and IL‐8 cytokines (Turroni et al., 2014).

The presence of *Bacteroides* and *Prevotella* in fecal slurry mixed with the following substrates (cooked quinoa, uncooked quinoa, and quinoa polysaccharides) (Figure 4) was responsible for the production of acetic and propionic acids (Table 1). On the other hand, the low amount of these acids after fermentation of FOS was induced by the decreased growth of these bacteria. A previous study showed that the production of these acids may decrease the synthesis of LDL cholesterol (Gómez et al., 2016), and we noticed that Clostridia in all fermentation substrates decreased from 59.62% to 14.60%–2%. It was demonstrated that Clostridia could not tolerate acidic conditions (Gibson & Roberfroid, 1995).

PCA was carried out to evaluate whether there were significant differences in the regulatory effects of different substrates (cooked, uncooked quinoa, and quinoa polysaccharides) on gut microbiota. As shown in Figure 5, different substrates showed distinct modulating effects on the fecal microbiota, which had been grouped separately. Both cooked and uncooked quinoa were closely grouped to each other according to their dispersal plots, which could be explained by the difference in their effects on the fecal microbiome.

Alpha diversity reflected the richness and diversity of microbial communities (Jiang et al., 2020). As shown in Table 2, a significant difference was observed between the fermented substrates and the blank. The value of Shannon and Simpson indexes of the cooked, uncooked quinoa, and quinoa polysaccharides was higher than those of positives control and lower than those of blank. In other words, these substrates changed the diversity of microbiota communities.

**TABLE 2:** α‐diversity indices of gut microbiota of the different substrate fermentation slurry (*n* = 3)

Sample	Observed species	Chao1	Shannon	Simpson
FOS	565.03 ± 21.17^c^	885.08 ± 24.22^ab^	3.33 ± 0.05^d^	0.69 ± 0.01^d^
QPs	620.33 ± 24.81^b^	913.40 ± 28.42^ab^	4.75 ± 0.08^b^	0.90 ± 0.01^b^
Cooked Q	546.73 ± 11.95^c^	848.47 ± 28.40^b^	3.65 ± 0.05^c^	0.82 ± 0.01^c^
Uncooked Q	543.10 ± 22.30^c^	817.96 ± 25.70^b^	3.67 ± 0.04^c^	0.80 ± 0.01^c^
Blank	739.60 ± 43.43^a^	946.31 ± 24.99^a^	6.07 ± 0.38^a^	0.94 ± 0.01^a^

Note

Results in each column are statistically different (ANOVA, LSD test, *p* <.05) with a > b > c > d.

Abbreviations: QPs, quinoa polysaccharides; FOS, Fructooligosaccharide.

The cooked quinoa was compared with uncooked quinoa, and the level of the bacterial microbiome was different when the seeds were cooked before fermentation. Statistical analysis showed that there were significant differences at the phylum level of Firmicutes and Proteobacteria. On the other hand, no difference has been detected at the phylum level of Bacteroidetes and Actinobacteria. Furthermore, at the genus level, *Prevotella*, *Mitsuokella*, and *Succinivibrion* had significant differences, but *Klebsiella* had no significant differences. We also found that in the media containing cooked quinoa, the total yield of short‐chain fatty acids was higher than that of uncooked quinoa, which was why we could predict that the effect of cooking marked fermentation produces more acids in the intestinal tract and cooking may make the components easier to be fermented or degraded. Previous research had shown that cooking decreased anti‐nutritional factors and improves protein content (Fawale et al., 2017; Castro‐Alba et al., 2019).

### 3.6 Correlation analysis of SCFAs and gut microbiota composition

**FIGURE 6:**
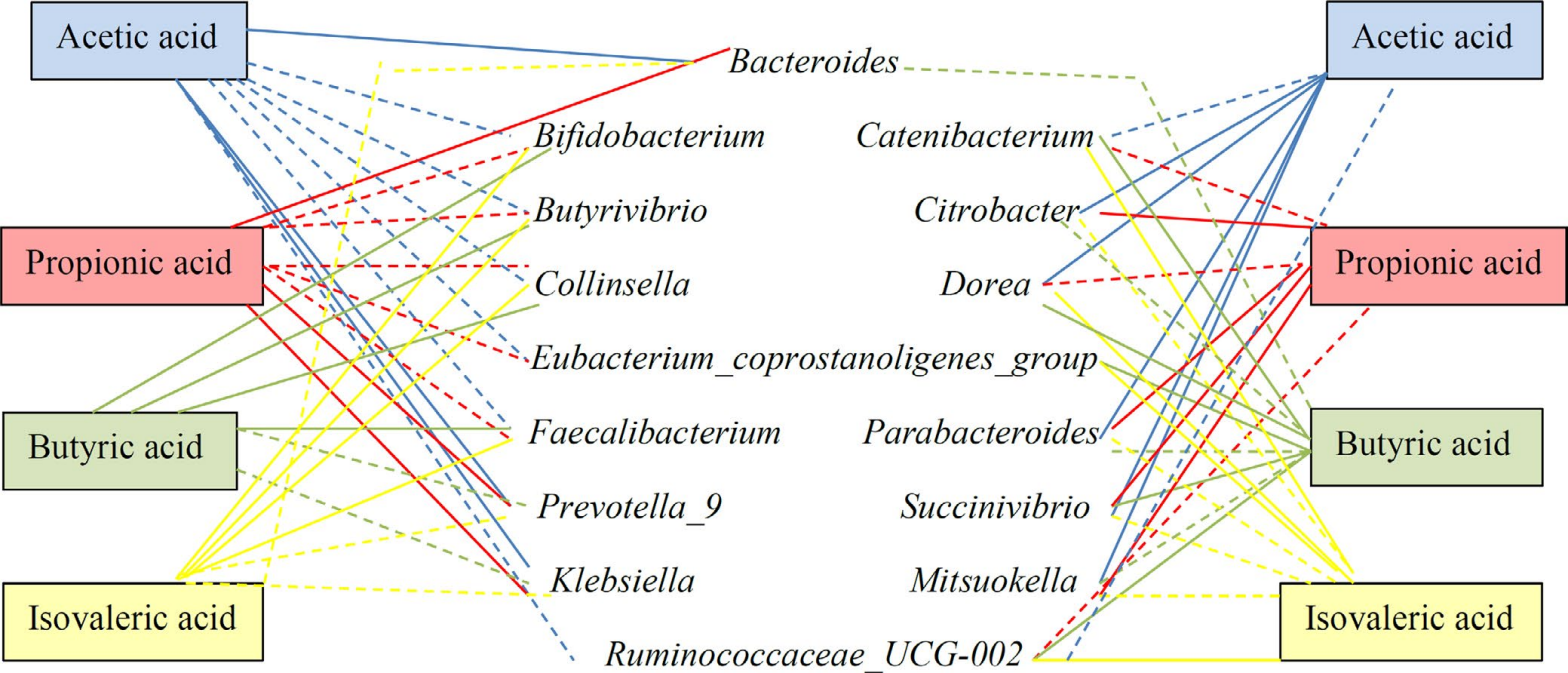
Correlations among specific bacteria and the formation of short‐chain fatty. The full line indicates a positive correlation; the dotted line indicates a negative correlation

As shown in Figure 6, there was a positive and negative correlation between the microbiota and the acids produced. We noticed that there was a positive correlation between acetic acid, propionic acid, and *Bacteroides*. This genus was commonly found in the human colon and stabilized its ecosystem by the catabolism of several polysaccharides. A previous study has shown that this genus produces propionic acid under different nutritional conditions (Adamberg et al., 2014). On the other hand, some components could alter the acid production by *Bacteroides*, such as some dietary supplements and *Aloe Vera* (Pogribna et al., 2008). We observed a positive correlation between acetic acid and *Prevotella*. The main metabolites of this genus were acetic acid and succinic acid (Hayashi et al., 2007). We also noticed a positive correlation between acetic acid and *Parabacteroides* genus, which was considered to be the major metabolites of this genus (Sakamoto & Benno, 2006). Moreover, there was a positive correlation between butyric acid and *Collinsella*, which was consistent with previous research where the authors isolated a *Collinsella* genus to produce butyric acid (Qin et al., 2019).

## 4 CONCLUSION

In this study, the in vitro effect of quinoa and quinoa polysaccharides on human gut health was investigated. Before fermentation, the cooked and uncooked quinoa were subjected to an in vitro digestion process. Polysaccharides were extracted from quinoa seeds. During the fermentation, the pH values of quinoa, quinoa polysaccharide, and FOS decreased, which was related to the production of short‐chain fatty acids, the main metabolites of the intestinal microbiota providing several beneficial functions for the epithelial cells and the immune system as well. The changes and diversities in the fecal microbiota after fermentation were analyzed at the phylum, genus, and class level. After the metagenomic analysis, it had been found that the quinoa substrates enhanced the growth of certain beneficial bacteria such as *Prevotella and Bacteroides*. Moreover, quinoa polysaccharides could be considered prebiotic due to their ability to increase *Bifidobacterium* and *Collinsella*. The PCA analysis revealed that cooked, uncooked quinoa and quinoa polysaccharides had significant differences of modulatory effect on gut microbiota compared with the blank and FOS, whereas the cooked and uncooked quinoa were grouped close to each other. These preliminary in vitro results encourage more exploration of quinoa and its intestinal health effects, including studies in vivo and other physiological parameters concerning human health.

## CONFLICTS OF INTEREST

The authors have declared no conflict of interest.

## AUTHOR CONTRIBUTIONS

**Zeyneb Hitache:** Conceptualization (supporting); Data curation (supporting); Formal analysis (lead); Investigation (lead); Visualization (lead); Writing‐original draft (lead). **Hairun Pei:** Conceptualization (supporting); Data curation (lead); Formal analysis (supporting); Funding acquisition (supporting); Project administration (lead); Writing‐review & editing (lead). **Xueli Cao:** Conceptualization (lead); Data curation (lead); Formal analysis (supporting); Funding acquisition (lead); Project administration (lead); Writing‐review & editing (supporting). **Wang Yuxin:** Conceptualization (supporting); Data curation (supporting); Formal analysis (supporting); Investigation (supporting); Visualization (supporting); Writing‐original draft (supporting). **Win Yumon:** Conceptualization (supporting); Data curation (supporting); Formal analysis (supporting); Investigation (supporting); Visualization (supporting); Writing‐original draft (supporting). **Lingxiao Gong:** Conceptualization (supporting); Data curation (supporting); Formal analysis (supporting); Investigation (supporting); Visualization (supporting); Writing‐original draft (supporting).

## ETHICAL APPROVAL

All experimental procedures were approved by the Institutional review board of Beijing Technology and Business University.
